# Efficacy and safety of sodium-glucose cotransporter 2 inhibitors initiation in patients with acute heart failure, with and without type 2 diabetes: a systematic review and meta-analysis

**DOI:** 10.1186/s12933-022-01455-2

**Published:** 2022-02-05

**Authors:** Husam M. Salah, Subhi J. Al’Aref, Muhammad Shahzeb Khan, Malek Al-Hawwas, Srikanth Vallurupalli, Jawahar L. Mehta, J. Paul Mounsey, Stephen J. Greene, Darren K. McGuire, Renato D. Lopes, Marat Fudim

**Affiliations:** 1 Division of Cardiology, Department of Medicine, University of Arkansas for Medical Sciences, Little Rock, AR USA; 2 Division of Cardiology, Department of Medicine, Duke University, 2301 Erwin Road, Durham, NC USA; 3 Duke Clinical Research Institute, Durham, NC USA; 4 Division of Cardiology, Department of Medicine, University Texas Southwestern, and Parkland Health and Hospital System, Dallas, TX USA

**Keywords:** Sodium-glucose cotransporter 2 inhibitors, Heart failure, Acute, Initiation, Outcomes, Systematic review, Meta-analysis

## Abstract

**Background:**

There is uncertainty and limited data regarding initiation of sodium-glucose cotransporter 2 (SGLT2) inhibitors among patients hospitalized with acute heart failure (AHF). This systematic review and meta-analysis aim to establish the efficacy and safety of SGLT2 inhibitors initiated in patients hospitalized for AHF.

**Methods:**

PubMed/Medline, Embase, and Cochrane library were searched using the following terms: (“sglt2" and "acute heart failure") and (“sglt2" and "worsening heart failure") from inception till November 15th, 2021 for randomized controlled trials (RCTs) comparing the efficacy and safety of initiating an SGLT2 inhibitor compared with placebo in patients with AHF. Major cardiovascular and diabetes scientific meetings in 2021 were also searched for relevant studies. Prespecified efficacy outcomes were all-cause mortality, rehospitalization for heart failure, and improvement in Kansas City Cardiomyopathy Questionnaire (KCCQ) scale score. Prespecified safety outcomes were acute kidney injury (AKI), hypotension, and hypoglycemia. Random effects odds ratio (OR) and mean difference with 95% confidence intervals (CIs) were calculated.

**Results:**

Three RCTs with a total of 1831 patients were included. Initiation of SGLT2 inhibitors in patients with AHF reduced the risk of rehospitalization for heart failure (OR 0.52; 95% CI [0.42, 0.65]) and improved Kansas City Cardiomyopathy Questionnaire scores (mean difference 4.12; 95% CI [0.1.89, 6.53]). There was no statistically significant effect for initiation of SGLT2 inhibitors in patients with AHF on all-cause mortality (OR 0.70; 95% CI [0.46, 1.08]). Initiation of SGLT2 inhibitors in patients with AHF did not increase the acute kidney injury (OR 0.76; 95% CI [0.50, 1.16]), hypotension (OR 1.17; 95% CI [0.80, 1.71]), or hypoglycemia (OR 1.51; 95% CI [0.86, 2.65]).

**Conclusion:**

Initiation of SGLT2 inhibitors in patients hospitalized for AHF during hospitalization or early post-discharge (within 3 days) reduces the risk of rehospitalization for heart failure and improves patient-reported outcomes with no excess risk of adverse effects.

**Supplementary Information:**

The online version contains supplementary material available at 10.1186/s12933-022-01455-2.

## Background

Results from randomized clinical outcomes trials (RCTs) and meta-analyses of patients with heart failure (HF) have shown that sodium-glucose cotransporter 2 (SGLT2) inhibitors improve cardiovascular (CV) outcomes in patients with chronic HF irrespective of diabetes status and across a wide spectrum of left ventricular ejection fraction [1–7]. In a meta-analysis of the EMPEROR-Reduced and DAPA-HF trials, both of which investigated SGLT2 inhibitors in patients with chronic HF with reduced ejection fraction (HFrEF), SGLT2 inhibitors resulted in a 13% reduction in all-cause death, 14% reduction in cardiovascular death, 31% reduction in first hospitalization for HF, and 38% reduction in adverse renal outcomes in these patients [8]. Given the robust evidence supporting the use of SGLT2 inhibitors in patients with HFrEF, the updated American and European guidelines in 2021 included SGLT2 inhibitors in the guideline-directed medical therapies (GDMT) for chronic HFrEF [9, 10]. In contrast, data regarding initiation of SGLT2 inhibitors among patients hospitalized with acute HF are more limited, and relative uncertainty regarding safety, tolerability, and efficacy with in-hospital initiation may cause clinicians to defer initiation of SGLT2 inhibitors to the outpatient setting [11]. This underscores the importance of evaluating the efficacy and safety of SGLT2 inhibitor initiation in patients hospitalized for AHF. Herein, the aim of this meta-analysis is to derive more reliable estimate of the efficacy and safety of SGLT2 inhibitors initiated in patients hospitalized for AHF.

## Method

PubMed/Medline, Embase, and Cochrane library from inception until November 15^th^, 2021, were searched using the following terms: (“sglt2" and "acute heart failure") and (“sglt2" and "worsening heart failure"). The major cardiovascular and diabetes meetings in 2021 (American Heart Association [AHA] Scientific Sessions 2021, American College of Cardiology Scientific Sessions 2021, European Society of Cardiology 2021 Congress, American Diabetes Association Scientific Sessions 2021, Heart Failure Society of America Scientific Meeting 2021, European Association for the Study of Diabetes 2021) were searched for relevant unpublished studies. The search was restricted in PubMed/Medline and Embase to clinical trials only using their advanced search tool. No other restrictions (e.g., sample size, follow-up period, or language) were applied. The prespecified selection criteria were: (1) randomized placebo-controlled clinical trials (RCTs); (2) the active arm of the trial included an SGLT2 inhibitor that was initiated in patients hospitalized with AHF; (3) trials reported selected prespecified efficacy and safety outcomes. Prespecified efficacy outcomes were all-cause mortality, rehospitalization for heart failure, and improvement in Kansas City Cardiomyopathy Questionnaire (KCCQ) scale score [12, 13]. Prespecified safety outcomes were acute kidney injury (AKI), hypotension, and hypoglycemia. The efficacy outcomes were chosen based on prior studies showing reduction in the risk of all-cause mortality and hospitalization for HF and improvement in KCCQ scale score in patients receiving SGLT2 inhibitors outside of the context of AHF [5, 14]. Safety outcomes were chosen based on tolerability concerns related to AKI, hypotension, and hypoglycemia in the setting of initiation of SGLT2 inhibitors in AHF [11].

Two investigators (H.M.S. and M.F.) conducted the study search, selection, and data abstracting. The same investigators independently appraised the potential risks of bias using the Cochrane Risk of Bias Tool 2.0 [15] and assessed the quality of the evidence using the GRADE (Grading of Recommendations Assessment, Development and Evaluation) approach (GRADEpro GDT) (https://gdt.gradepro.org/app/handbook/handbook.html#h.svwngs6pm0f2).

A random effects model meta-analysis was conducted using Mantel–Haenszel odds ratios (ORs) and the associated 95% confidence intervals (CIs) to assess all-cause mortality, rehospitalization for HF, worsening HF, AKI, hypotension, and hypoglycemia, and the inverse variance of weighted mean difference and associated 95% CIs were used to assess changes in KCCQ. The Cochrane Q statistic, and Higgins and Thompsons' I^2^ were used to evaluate heterogeneity, and the GRADE (Grading of Recommendations Assessment, Development and Evaluation) approach to assess the certainty of the evidence was used. Heterogeneity was considered to be low if I^2^ is less than 25%, moderate if I^2^ is between 25 and 75%, and high if I^2^ is greater than 75%. Assessment for publication bias was limited due to the low number of studies (< 10) each with limited statistical power. A sensitivity analysis was done by excluding the results of the unpublished EMPULSE trial. All analyses were done using the Review Manager software (version 5.4.1, The Cochrane Collaboration, 2020).

## Results

**Fig. 1:**
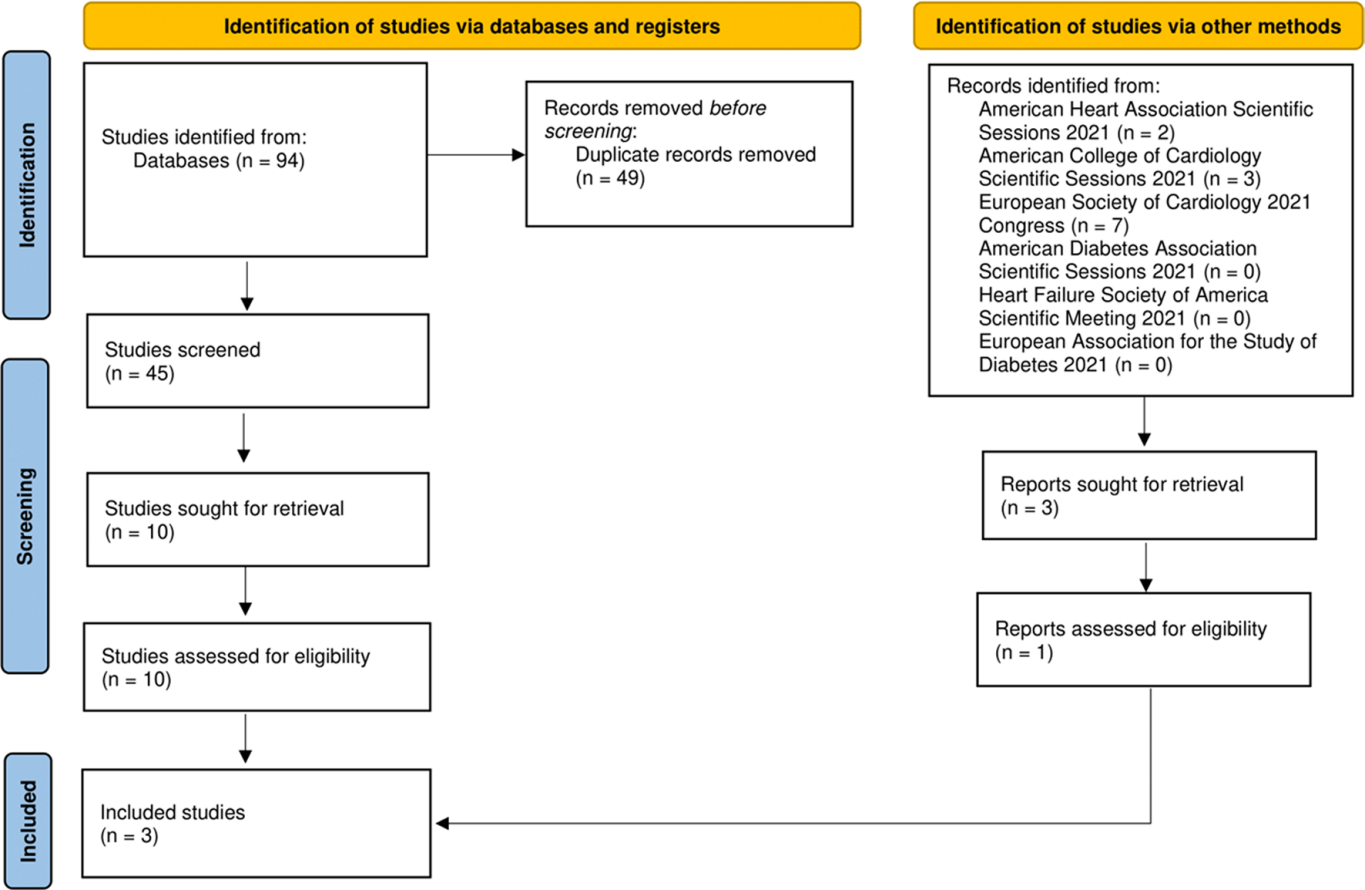
Preferred Reporting Items for Systematic Reviews and Meta-Analyses (PRISMA) flow diagram for the included studies

**Fig. 2:**
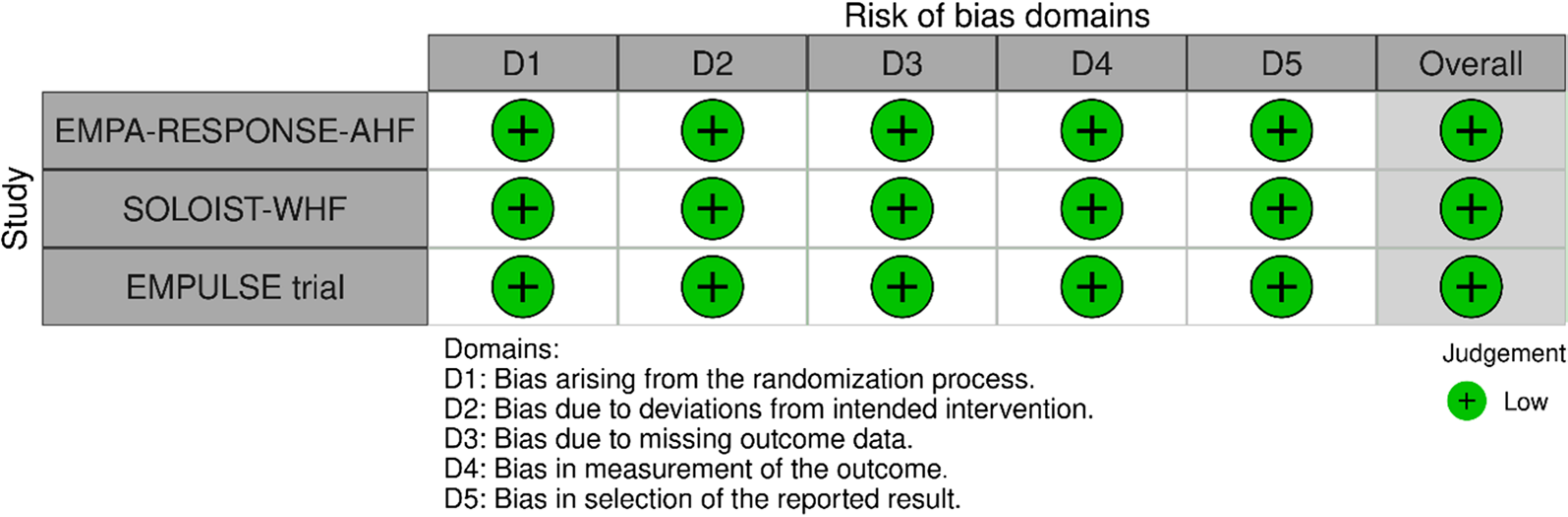
Risk of bias assessment using the Risk of Bias 2.0 tool. As the EMPUSLE trial results are not published yet, its risk of bias assessment was done based on its published design and the publicly available American Heart Association 2021 presentation

The initial search yielded 106 studies (94 from databases and 12 from scientific meetings). After applying the inclusion criteria, only 3 trials with a total of 1831 patients were included (two from databases and 1 from the AHA Scientific Sessions 2021; Fig. 1) [3, 16]. Follow-up periods differed between the included studies (60 days in the EMPA-RESPONSE-AHF, 90 days in the EMPULSE trial, and a median of 9 months in the SOLOIST-WHF trial). Characteristics of the included studies are summarized in Table 1. All studies were considered to have a low risk of bias (Fig. 2). Due to the lack of power with less than 10 studies included, we did not seek to evaluate for publication bias using the funnel plot approach [17]. Certainty of evidence assessment is summarized in Additional file 1: Table S1. Risk of bias and certainty of evidence for the unpublished EMPULSE trial were assessed based on the published study design and the AHA Scientific Sessions 2021 presentation [18].

**Table 1:** Characteristics of the included studies

Trial	EMPA-RESPONSE-AHF	SOLOIST-WHF	EMPULSE trial
Year	2020	2021	2021
SGLT2 inhibitor agent	Empagliflozin	Sotagliflozin	Empagliflozin
Type of patients	Patients with acute heart failure regardless of diabetes status	Patients with acute heart failure and type 2 diabetes	Patients with acute heart failure regardless of diabetes status
Participants (N)	79	1,222	530
SGLT2 inhibitor group (N)	40	608	265
Placebo group (N)	39	614	265
Age, years (mean)	76	69	71
Women (%)	33%	34%	33%
Race	Empagliflozin group had 100% Whites, whereas placebo group had 95% Whites and 5% others	Sotagliflozin group had 93.3% Whites, 4.1% Blacks, and 1.3% Asians, whereas placebo group had 93.2% Whites, 4.1% Blacks, and 1.1% Asians	Not reported in the American Heart Association 2021 presentation
Diabetes	Empagliflozin group: 38% Placebo group: 28%	100% in both groups	Empagliflozin group: 46.8% Placebo group: 43.8%
Hypertension	Empagliflozin group: 68% Placebo group: 56%	Not reported	Empagliflozin group: 77.4% Placebo group: 83.4%
Myocardial infarction	Empagliflozin group: 30% Placebo group: 38%	Not reported	Empagliflozin group: 24.9% Placebo group: 23.4%
Beta-blocker use	Empagliflozin group: 70% Placebo group: 66%	Sotagliflozin group: 92.8% Placebo group: 91.4%	Not reported in the AHA presentation
ACEi use	Empagliflozin group: 40% Placebo group: 47%	Sotagliflozin group: 41.8% Placebo group: 39.3%	Not reported in the AHA presentation
ARB use	Empagliflozin group: 5% Placebo group: 3%	Sotagliflozin group: 40.3% Placebo group: 44%	Not reported in the AHA presentation
ARNI use	Empagliflozin group: 5% Placebo group: 3%	Sotagliflozin group: 15.3% Placebo group: 18.2%	Not reported in the AHA presentation
MRA use	Empagliflozin group: 48% Placebo group: 45%	Sotagliflozin group: 66.3% Placebo group: 62.7%	Not reported in the AHA presentation
Follow-up	60 days	9 months	90 days

**Fig. 3:**
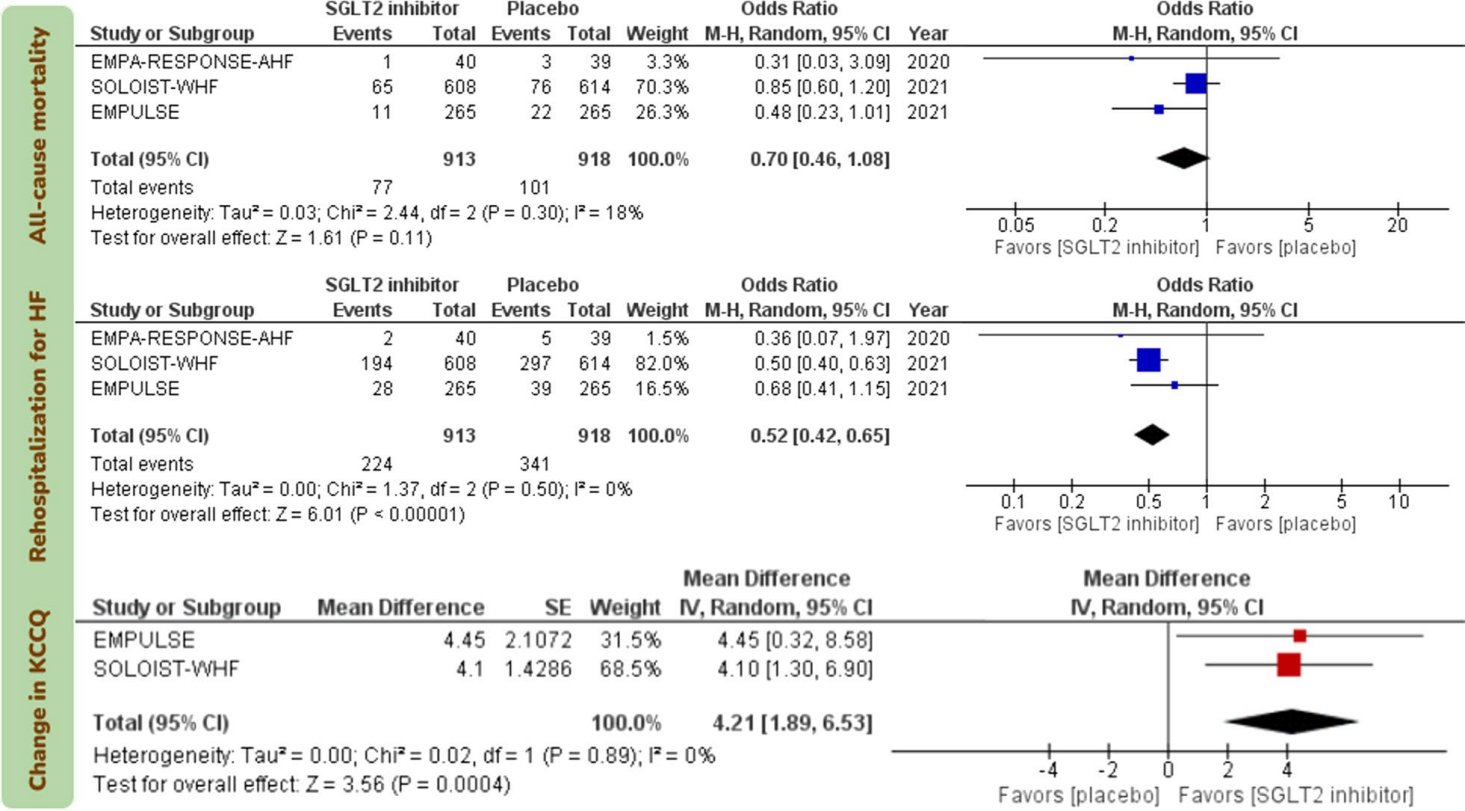
Forest plots examining the efficacy endpoints of sodium-glucose cotransporter 2 inhibitors initiation in patients with acute heart failure. Events in the analyses represent total number of events rather than time-to-event endpoint data. SGLT2: sodium-glucose cotransporter 2; HF: heart failure; KCCQ: Kansas City Cardiomyopathy Questionnaire; M–H: Mantel–Haenszel; CI: confidence interval

Initiation of SGLT2 inhibitors in patients with AHF reduced the risk of rehospitalization for HF (OR 0.52; 95% CI [0.42, 0.65]; I^2^ = 0%; certainty: high) and improved KCCQ scores, which were measured 4 months after treatment in the SOLOIST-WHF trial and 90 days after treatment in the EMPUSLE trial (mean difference 4.12; 95% CI [0.1.89, 6.53]; I^2^ = 0%; certainty: high; Fig. 3).

**Fig. 4:**
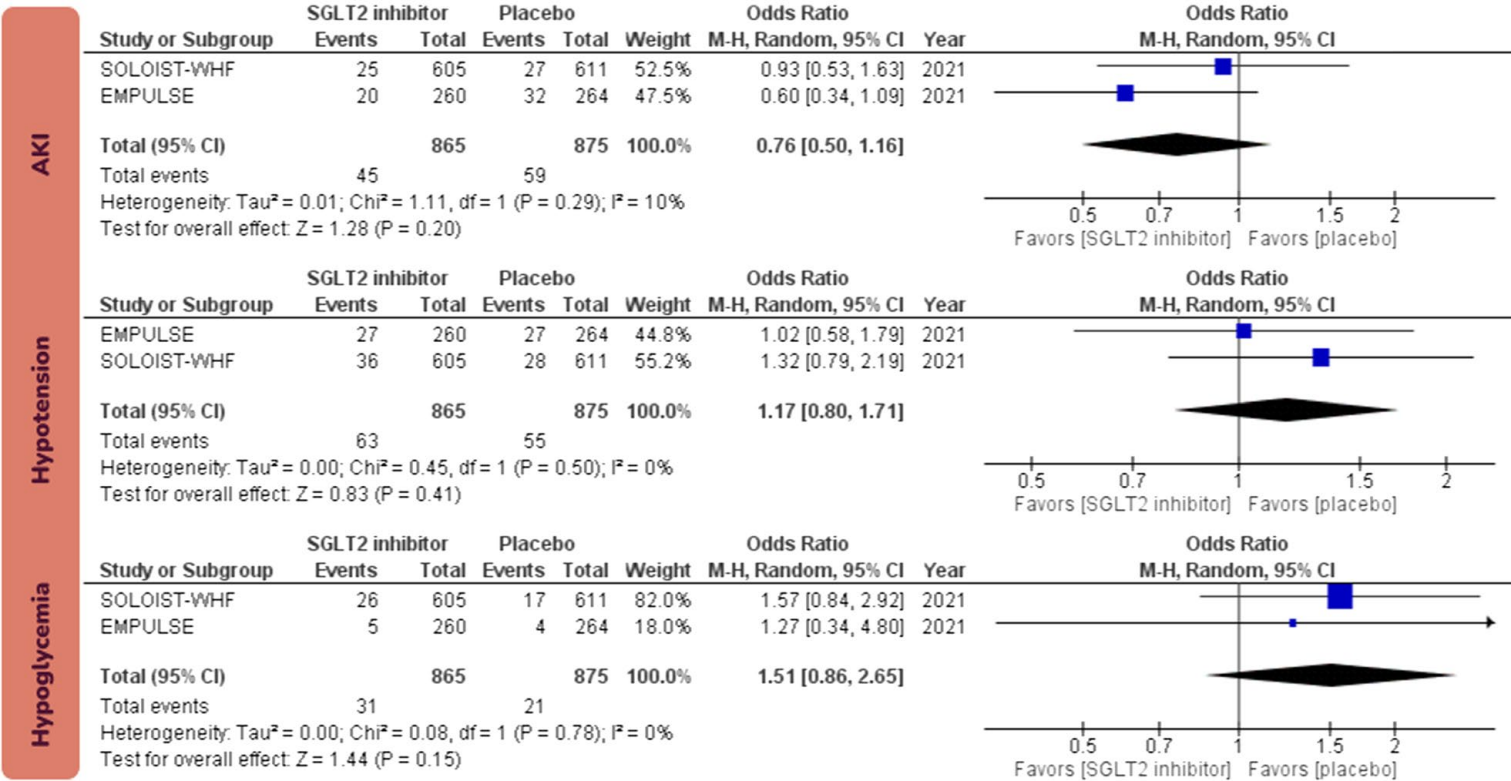
Forest plots examining the safety endpoints of sodium-glucose cotransporter 2 inhibitors initiation in patients with acute heart failure. Events in the analyses represent total number of events rather than time-to-event endpoint data. SGLT2: sodium-glucose cotransporter 2; HF: heart failure; AKI: acute kidney injury; M–H: Mantel–Haenszel; CI: confidence interval

**Fig. 5:**
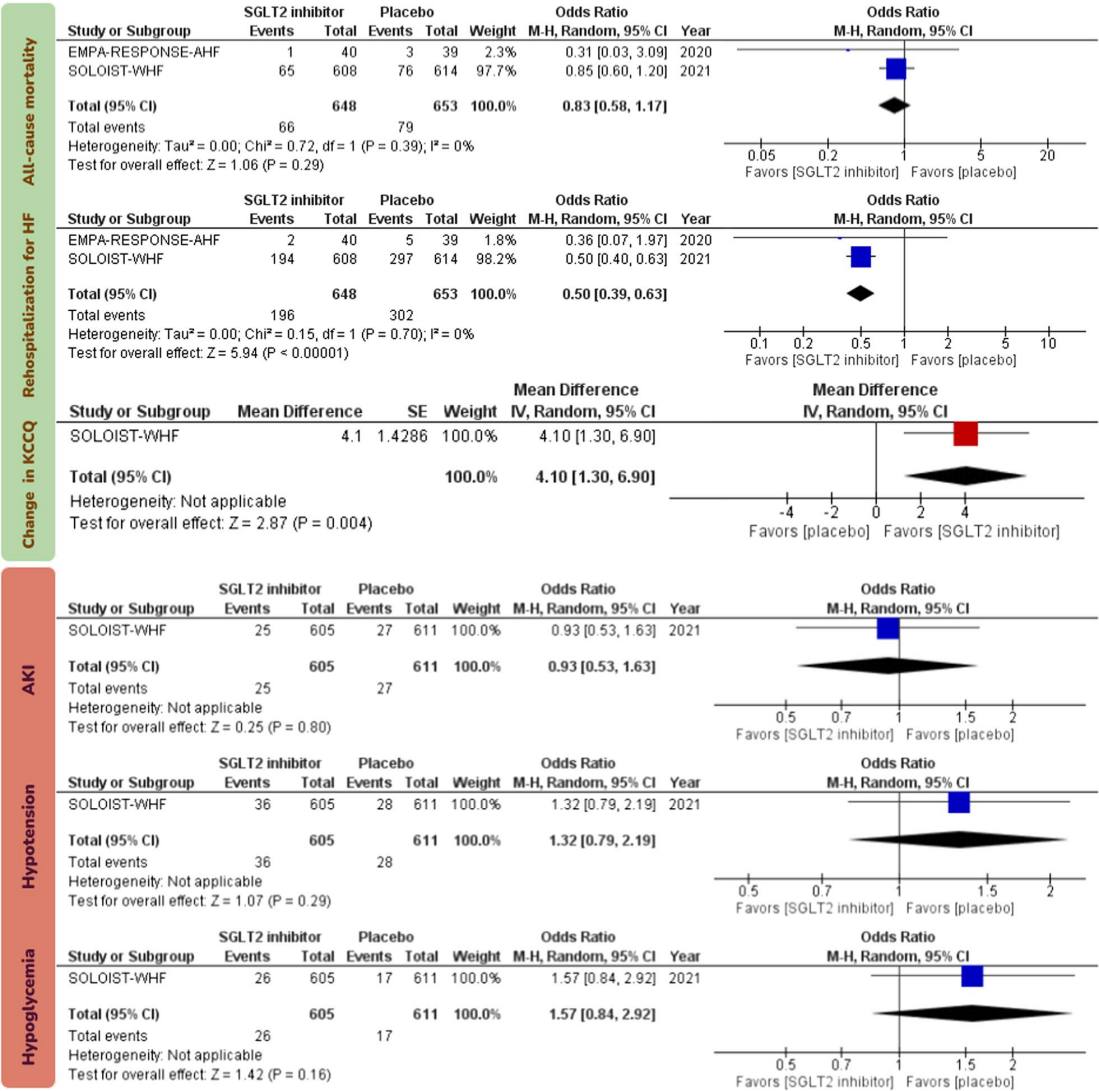
Results of sensitivity analyses excluding the results of the unpublished EMPUSLE trial

There was no statistically significant effect for initiation of SGLT2 inhibitors in patients with AHF on all-cause mortality (OR 0.70; 95% CI [0.46, 1.08]; I^2^ = 18%; certainty: high). Initiation of SGLT2 inhibitors in patients with AHF did not increase the risk AKI (OR 0.76; 95% CI [0.50, 1.16]; I^2^ = 10%; certainty: high), hypotension (OR 1.17; 95% CI [0.80, 1.71]; I^2^ = 0%; certainty: high), or hypoglycemia (OR 1.51; 95% CI [0.86, 2.65]; I^2^ = 0%; certainty: high; Fig. 4). Sensitivity analysis by excluding the unpublished EMPULSE trial from the analysis yielded consistent results (Fig. 5).

## Discussion

The main findings of this systematic review and meta-analysis are that initiation of SGLT2 inhibitors in patients with AHF during hospitalization or early post-discharge carries 48% lower odds of rehospitalization for HF and significant improvements in patient-reported outcomes (as measured by KCCQ), without excess risk of AKI, hypotension, or hypoglycemia.

Previous studies have shown that failure to initiate GDMT at discharge from a HF hospitalization is associated with a considerable risk that this therapy will not be started during subsequent follow-ups or will be started with significant delay [19, 20]. In contrast, patient discharged on GDMT are more likely to be adherent with these therapies following their discharge [20]. Hesitation to start SGLT2 inhibitors in patients hospitalized with AHF stems from concerns of adverse effects in these patients with a clinically tenuous status (e.g., hypotension, AKI, and hypoglycemia) [11]. The present meta-analysis demonstrates that initiating GDMT with SGLT2 inhibitor in patients with AHF during their hospitalizations or shortly thereafter (within 3 days of discharge) is safe and significantly reduces the risk of rehospitalization following discharge. These findings, in the light of the previous studies [19, 20], suggest that failure to initiate SGLT2 inhibitors in patients with AHF would be a significant missed opportunity that would prevent rehospitalization, improve quality of life, and jeopardize the opportunity to address the rising trend of hospitalization for HF in the recent years [21, 22].

In addition to the mechanisms driving the chronic cardioprotective benefits of SGLT2 inhibitors (e.g., optimizing cardiac energy metabolism, anti-inflammatory effect, inhibition of the sympathetic nervous system, prevention of ischemia and reperfusion injury, reduction in oxidative stress) [23], initiation of SGLT2 inhibitors in patients with AHF can likely lead to a rapid (as early as day 1 of treatment) and sustained volume unloading and improvement of left ventricular filling pressure and diastolic function [24], which may contribute to the significant reduction in the risk of rehospitalization for HF observed in the present meta-analysis. It is unclear if these benefits in AHF are mediated by a diuretic role for SGLT2 inhibitors; in an analysis of the EMPEROR-Reduced trial, empagliflozin reduced the combined risk of CV mortality or hospitalization for HF in patients with and without recent volume overload (i.e., 4 weeks before enrollment) with no difference between the groups [25]. Further, changes in body weight correlated poorly with changes in natriuretic peptides or hematocrit, and there were no observed significant changes in serum sodium values [25]. These findings suggest a less dominant diuretic role for SGLT2 inhibitors in their cardioprotective mechanisms. Interestingly, in patients with stable chronic HF, the empagliflozin-induced increase in ketone bodies as assessed by beta-hydroxybutyrate causes an attenuation of the beneficial effects of empagliflozin on blood pressure and vascular parameters [26]. While these findings have not yet been confirmed in patients with AHF, such findings may suggest a less pronounced role for afterload reduction as a mechanism driving volume unloading and optimization of left ventricular filling pressure following initiation of SGLT2 inhibitors.

There are several limitations to these meta-analyses that should be noted. First, published summary data rather than individual patient level data were used. Individual patient level data meta-analyses allow for line-by-line patient data collection from the included studies as opposed to only analyzing the measure of effect in published summary data meta-analyses [27]. They also allow for a more consistent identification of the exposure and outcomes across the studies and better adjustment for confounders to minimize heterogeneity [27]. We observe low heterogeneity in all the analyses presented in the current meta-analysis. Second, while the EMPULSE trial used the KCCQ-total symptoms score to measure patient-reported outcomes, the SOLOIST-WHF trial used the KCCQ-12, which is the shorter version. However, both scores are scaled similarly (0–100), and KCCQ-12 strongly correlates with the original scale scores with comparable validity, reliability, responsiveness, and prognostic value [13]. Third, the included trials had variable follow-up periods ranging from 60 days as in EMPA-RESPONSE-AHF trial to more than 9 months as in the SOLOIST-WHF trial; while 60 days can be considered a relatively short follow-up period, the main aim of these meta-analyses was to establish the efficacy and safety of SGLT2 inhibitor initiation in the acute setting of HF exacerbation, and for which this follow-up period should be fairly adequate. Fourth, while both EMPA-RESPONSE-AHF and EMPULSE initiated treatment with SGLT2 prior to discharge in all patients hospitalized for AHF, the SOLOIST-WHF allowed inclusion of patients receiving the first dose of SGLT2 inhibitor up to 3 days after discharge.

## Conclusion

Initiation of SGLT2 inhibitors in patients hospitalized for AHF before discharge or shortly after (within 3 days of discharge) resulted in a reduction in the risk of rehospitalization and improved patient-reported outcomes with no apparent increase in the risk of adverse effects.

## Supplementary Information

**Additional file 1: Table S1.** Certainty of evidence assessment using GRADE (Grading of Recommendations Assessment, Development and Evaluation) approach.

## Data Availability

All the data used to generate this meta-analysis is publicly available.

## Abbreviations

SGLT2: Sodium-glucose cotransporter 2
GDMT: Guide-directed medical therapy
AHF: Acute heart failure
OR: Odds ratio
CI: Confidence interval
GRADE: Grading of Recommendations Assessment, Development and Evaluation
AKI: Acute kidney injury
KCCQ: Kansas City Cardiomyopathy Questionnaire
