# Supplementary material for: Efficacy and safety of sodium-glucose cotransporter 2 inhibitors initiation in patients with acute heart failure, with and without type 2 diabetes: a systematic review and meta-analysis
Source: Cardiovasc Diabetol. 2022 Feb 5;21:20. doi: 10.1186/s12933-022-01455-2 (PMC8817537; doi:10.1186/s12933-022-01455-2)
Supplement: Supplementary file 1 — Additional file 1: Table S1. Certainty of evidence assessment using GRADE (Grading of Recommendations Assessment, Development and Evaluation) approach. [file 12933_2022_1455_MOESM1_ESM.docx]

**Supplemental File**

**Table 1.** Certainty of evidence assessment using GRADE (Grading of Recommendations Assessment, Development and Evaluation) approach.

| **Certainty assessment** | | | | | | | **№ of patients** | | **Effect** | | **Certainty** |  |
| --- | --- | --- | --- | --- | --- | --- | --- | --- | --- | --- | --- | --- |
| **№ of studies** | **Study design** | **Risk of bias** | **Inconsistency** | **Indirectness** | **Imprecision** | **Other considerations** | **AHF and SGLT2i** | **placebo** | **Relative (95% CI)** | **Absolute (95% CI)** |  |  |
| **HF rehospitalization** | | | | | | | | | | | | |
| 3 | randomised trials | not serious | not serious | not serious | not serious | none | 224/913 (24.5%) | 341/918 (37.1%) | **OR 0.52** (0.42 to 0.65) | **136 fewer per 1,000** (from 173 fewer to 94 fewer) | ⨁⨁⨁⨁ High |  |
| **All-cause mortality** | | | | | | | | | | | | |
| 3 | randomised trials | not serious | not serious | not serious | not serious | none | 77/913 (8.4%) | 101/918 (11.0%) | **OR 0.70** (0.46 to 1.08) | **30 fewer per 1,000** (from 56 fewer to 8 more) | ⨁⨁⨁⨁ High |  |
| **Improvement in KCCQ** | | | | | | | | | | | | |
| 2 | randomised trials | not serious | not serious | not serious | not serious | none | 0 | 0 | - | MD **4.21 higher** (1.89 higher to 6.53 higher) | ⨁⨁⨁⨁ High |  |
| **Acute kidney injury** | | | | | | | | | | | | |
| 2 | randomised trials | not serious | not serious | not serious | not serious | none | 45/865 (5.2%) | 59/875 (6.7%) | **OR 0.76** (0.50 to 1.16) | **15 fewer per 1,000** (from 33 fewer to 10 more) | ⨁⨁⨁⨁ High |  |
| **Hypoglycemia** | | | | | | | | | | | | |
| 2 | randomised trials | not serious | not serious | not serious | not serious | none | 31/865 (3.6%) | 21/875 (2.4%) | **OR 1.51** (0.86 to 2.65) | **12 more per 1,000** (from 3 fewer to 37 more) | ⨁⨁⨁⨁ High |  |
| **Hypotension** | | | | | | | | | | | | |
| 2 | randomised trials | not serious | not serious | not serious | not serious | none | 63/865 (7.3%) | 55/875 (6.3%) | **OR 1.17** (0.80 to 1.71) | **10 more per 1,000** (from 12 fewer to 40 more) | ⨁⨁⨁⨁ High |  |

**CI:** confidence interval; **MD:** mean difference; **OR:** odds ratio
